# Electrochemical and Stress Corrosion Mechanism of Submarine Pipeline in Simulated Seawater in Presence of Different Alternating Current Densities

**DOI:** 10.3390/ma11071074

**Published:** 2018-06-25

**Authors:** Wei Wu, Yue Pan, Zhiyong Liu, Cuiwei Du, Xiaogang Li

**Affiliations:** Corrosion and Protection Center, University of Science and Technology Beijing, Beijing 100083, China; wuwei19910117@126.com (W.W.); panyue940130@xs.ustb.edu.cn (Y.P.); dcw@ustb.edu.cn (C.D.); lixiaogang99@263.net (X.L.)

**Keywords:** pipeline steel, alternating current, polarization, stress corrosion

## Abstract

In this study, electrochemical measurements, immersion tests, and slow strain rate tensile (SSRT) tests were applied to investigate the electrochemical and stress corrosion cracking (SCC) behavior of X70 steel in simulated seawater with the interference of different alternating current (AC) densities. The results indicate that AC significantly strengthens the cathodic reaction, especially the oxygen reduction reaction. Simultaneously, hydrogen evolution reaction occurs when the limiting diffusion current density of oxygen reaches, and thus, *i*_corr_ sharply increases with the increase in AC density. Additionally, when AC is imposed, the X70 steel exhibits higher SCC susceptibility in the simulated seawater, and the susceptibility increases with the increasing AC density. The SCC mechanism is controlled by both anodic dissolution (AD) and hydrogen embrittlement (HE) with the interference of AC.

## 1. Introduction

Due to the rapid development of oil and gas resources inshore, pipeline steels are widely used in the exploitation and transportation of petroleum gas. Pipeline steels, which may face structural and residual stress in weld joints, tend to be corroded in seawater, and damaged by heavy loads. These synergetic effects make these structures brittle to stress corrosion cracking (SCC), as a result, environmental pollution and safety accidents may occur. Although there are few SCC failures acknowledged in marine environments [1,2], plenty of accidents caused by SCC in onshore oil and gas pipelines have raised concerns [3,4,5,6]. In general, SCC in pipelines takes place when the wearing and peeling of the coating reveals open defects, which facilitates the contact of corrosive media and the metal surface. As a result, different mechanisms of SCC are generated, which are primarily classified as high-pH SCC [7,8,9] and near-neutral pH SCC [10,11]. High-pH SCC always occurs where cathodic protection (CP) conditions are strong, the pH is greater than nine, and the SCC crack propagation is intergranular (IGSCC). By contrast, relatively weaker CP or CP-free conditions bring about the occurrence of near-neutral pH SCC, which usually appears when the pH is less than 7.5 [12]. Undoubtedly, offshore pipelines also have these problems with corrosion. Once the coatings on the surface of pipelines are destroyed, the permeation of seawater into the gaps will promote the peeling of coatings and create complicated corrosion environments. Therefore, high-pH or near-neutral pH environments rich in Cl^−^ is generated where coatings fail and open defects exist. Once a pipeline has been in service for an extended period of time, SCCs are easily initiated in these environments.

What is more, the leakage and perforation of petroleum pipelines in the soil environment caused by corrosion with the interference of alternating current (AC) have occurred in many places worldwide [13,14,15,16]. Many studies concentrated on this corrosion behavior [17,18,19,20,21], and their results suggested that high AC density induced local corrosion, particularly pitting corrosion. It is well-known that pitting corrosion is one of the sensitivity factors leading to SCC [22], which can result in the initiation of microcracks during the stress corrosion [23,24]. It is determined that AC can directly increase the susceptibility of pipeline steel to SCC, which transforms the relevant SCC mechanism from anodic dissolution (AD) to a mixture of AD and hydrogen embrittlement (HE) [25]. Thus, in the presence of AC, the SCC of pipelines has become more serious, and the interaction between AC and SCC has become an important area of research [26,27].

Additionally, AC may have a serious impact on the SCC of the submarine pipelines. With the rapid growth of economy, additional traffic facilities, buildings, and inshore or offshore electricity facilities are likely to develop. These developments lead to additional CP, communication, electric transmission, and transformation equipment, which can simultaneously generate a large amount of AC stray current in offshore districts. In sea field, the influence of AC stray current is more severe due to the higher conductivity, intensity of alternating electric field, and larger sphere of influence. Kim et al. [28,29] studied the corrosion behavior of carbon steel under different AC current densities in simulated seawater and proved that AC could accelerate corrosion even with CP and, to some extent, result in pitting corrosion and SCC. Therefore, in inshore or offshore districts with AC, a higher risk of SCC in pipelines may exist. However, little attention has been devoted to investigating this occurrence. For these reasons, studying the SCC behavior and mechanism with the interference of AC under the marine conditions are of great importance. 

In this work, electrochemical measurement, U-bend sample immersion, and slow strain rate tensile (SSRT) tests were applied to investigate the SCC behavior of an API X70 pipeline steel in simulated seawater and its variation with AC densities, and further to explore the SCC mechanism of the submarine pipelines under the interference of AC.

## 2. Experimental

### 2.1. Material and Solution

**Figure 1:**
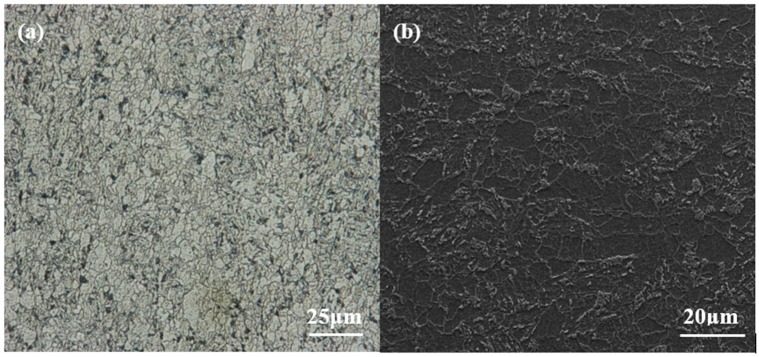
Microstructural images of the X70 pipeline steel: (**a**) metallograph and (**b**) scanning electron microscope (SEM) diagram.

All the specimens were taken from an API X70 steel plate. The main chemical compositions (in wt %) of X70 steel were as follows: C 0.073, Si 0.24, Mn 1.48, S 0.0022, P 0.0035, Al 0.029, Cu 0.21, Fe balance. The microstructure of the steel was observed with KEYENCE VHX-2000 stereomicroscope and QUANTA 250 scanning electron microscope (SEM), respectively as shown in Figure 1. The microstructure of the X70 steel is primarily comprised of bainite and ferrite, with martensite/austenite (M/A) islands among the grain boundaries. The experimental solution was simulated seawater with the compositions of (g/L) NaCl 26.726, MgCl_2_ 2.260, MgSO_4_ 3.248, CaCl_2_ 1.153, KCl 0.721, NaHCO_3_ 0.198, and its pH was adjusted to approximately 8.10 (close to the pH of seawater) with 0.01 mol/L NaOH solution. The solution was aerated for all the performed experiments.

### 2.2. Electrochemical Measurements

**Figure 2:**
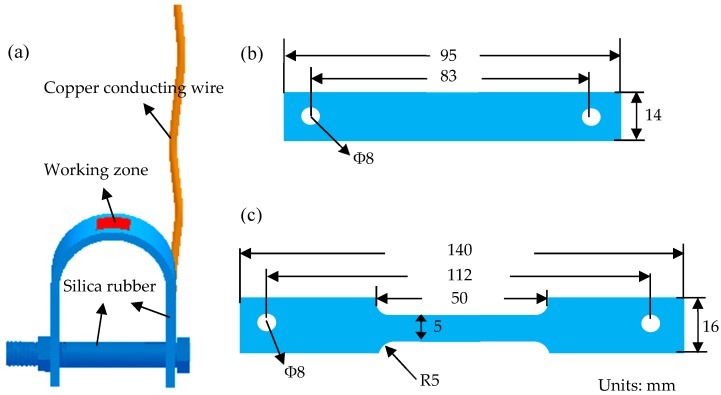
(**a**) Schematic of the U-bend samples for electrochemical measurements and immersion tests; (**b**) and (**c**) are the geometry of the U-bend and SSRT samples, respectively. The thicknesses of the U-bend and slow strain rate tensile (SSRT) samples are 2 mm.

The electrochemical specimens consisted of stressed specimens and non-stress specimens, where the non-stress specimens were 10 × 10 mm^2^ square, and the stressed specimens were U-bend specimens prepared according to Chinese National Standards GB/T 15970. The red area was the working zone under the maximum strain, as shown in Figure 2a, and the yellow area was the copper conducting wire. The other portions of the specimens were insulated by a thin, compact layer of silica rubber. The geometry of the U-bend sample is shown in Figure 2b. Before testing, the specimens were ground with emery paper, washed by de-ionized water and acetone, and blow-dried. 

**Figure 3:**
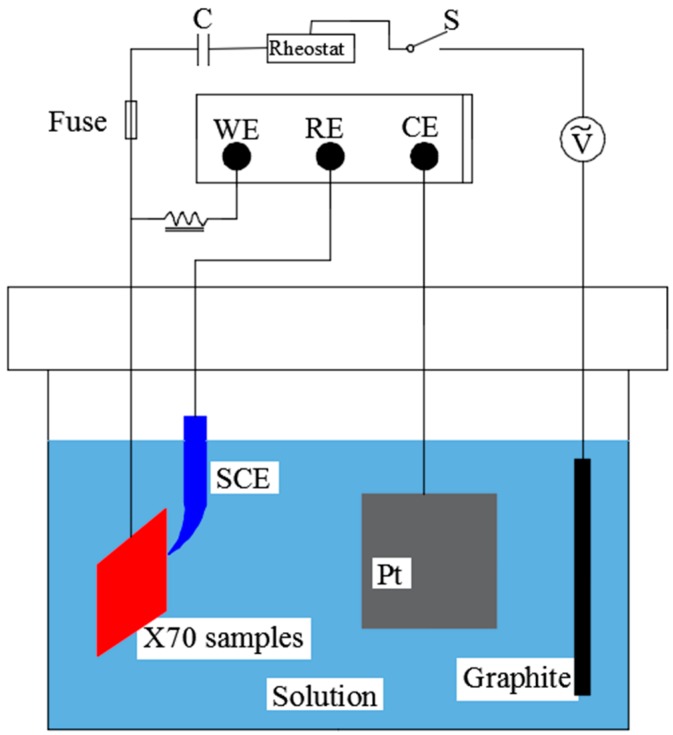
Schematic of the setup for electrochemical measurements.

Electrochemical measurements were conducted on a P2273 Electrochemical Workstation. Figure 3 shows the experimental setup, which consisted of an electrochemical measurement circuit and a simulated AC circuit. The electrochemical measurement circuit was assembled as a three-electrode cell: a platinum gauge was the counter electrode (CE), a saturated calomel electrode (SCE) served as the reference electrode (RE), and the specimen functioned as a working electrode (WE). A graphite gauge acted as the counter electrode in the AC circuit, in which AC signals were provided by an ATF05C AC signal generator. An inductor and a capacitor were prepared to maintain the tests normally, and the value of AC density was measured by a multimeter.

To study how the AC density influences the electrochemical behavior of X70 steel, different densities (0 A/m^2^, 10 A/m^2^, 30 A/m^2^, 50 A/m^2^, 100 A/m^2^) with a constant frequency of 50 Hz of the sine wave ACs were imposed. After a steady value of corrosion potential was achieved, the potentiodynamic tests were conducted with the range from −0.5 V to 0.7 V (vs. the steady value of corrosion potential) at the sweeping rate of 0.5 mV/s under the AC application. All of the experiments were performed at room temperature (~25 °C) and repeated three times.

### 2.3. Immersion Tests

The U-bend specimens used for immersion were also shown in Figure 2a. For testing, the specimen was immersed into the solution and functioned as the WE. A graphite gauge served as the CE. Sine wave AC signals were provided by an ATF05C AC signal generator, and a multimeter was used to measure the specific value of the current. The corrosion products on the top areas of U-bend specimens were regularly observed by stereomicroscope when immersing periods were 3 days, 7 days, 15 days, and 30 days. After immersion for 30 days, the tops of the U-bend samples were cut out to examine the surface morphology. The corrosion products were cleaned with the descaling liquid (500 mL of HCl + 500 mL of H_2_O + 5 g of hexamethylene tetramine), and relevant corrosion morphology was observed by SEM.

### 2.4. SSRT Tests

The specimens were prepared according to Chinese National Standards GB/T 15970, whose geometry is shown in Figure 2c. Prior to testing, the steels were orderly ground with 60# to 2000# emery paper along the tensile direction and washed with ethyl alcohol. The SSRT tests were finished with a strain rate of 1 × 10^−6^ s^−1^ through a WDML-30KN Materials Test System. A platinum gauge was selected as the CE to apply the interference of AC current, and the tensile specimen functioned as the WE. Once the complete experimental setup was established, AC signals were imposed, which would continue until the specimen cracked. Each test was conducted three times to ensure the repeatability and reliability.

After the SSRT tests, to investigate the susceptibility of steel to SCC under various densities of AC, the reduction in area (*I*_Ψ_) and the elongation loss rate (*I_δ_*) were calculated using the following equations:

(1) $$I_{\Psi }=(1-\frac{\Psi_{E}}{\Psi_{0}})\times 100\%$$

and

(2) $$I_{\delta }=(1-\frac{\delta_{E}}{\delta_{0}})\times 100\%$$

where Ψ*_E_* and *δ_E_* are the percentage reduction of area and percentage elongation of steel measured at the solution, respectively; and Ψ_0_ and *δ*_0_ are the percentage reduction of area and percentage elongation of steel measured in air, respectively.

## 3. Results

### 3.1. Potentiodynamic Polarization Tests

**Figure 4:**
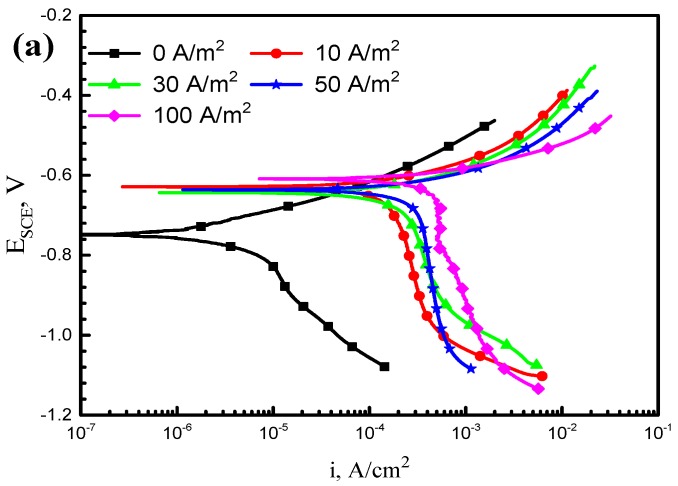

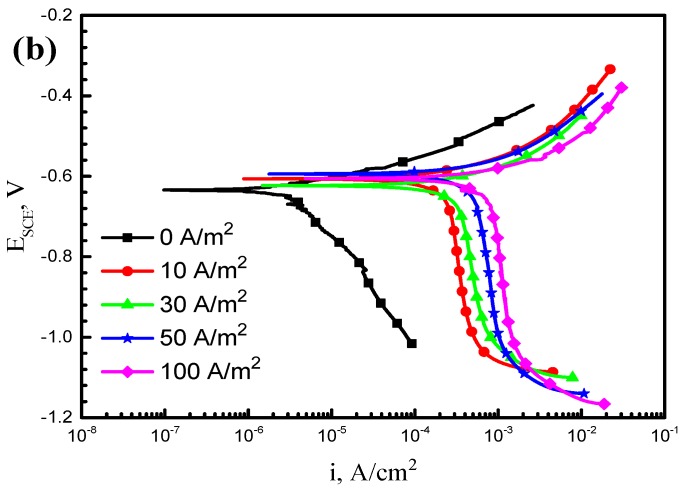
Polarization curves of the X70 steel tested at various AC densities in simulated seawater: (**a**) non-stress samples and (**b**) U-bend samples.

Figure 4 shows the electrochemical behavior of the X70 steel at different AC densities in simulated seawater. As shown in Figure 4a, after the AC signals were applied, the curve shows an obvious right shift and the corrosion current density increased considerably. Moreover, as the AC density was gradually elevated from 10 A/m^2^ to 100 A/m^2^, the curve successively shifted toward the right, the corrosion current density (*i*_corr_) rose, and the zero-current potential roughly remained constant. The anodic Tafel slope was much lower than the cathodic Tafel slope, that is to say, the electrochemical reaction was mainly controlled by the cathodic process. Distinctly, AC promotes the cathodic process in simulated seawater, and hydrogen evolution reactions may occur in addition to the oxygenation reaction, indicating that the mechanism of cathodic reactions may change.

**Figure 5:**
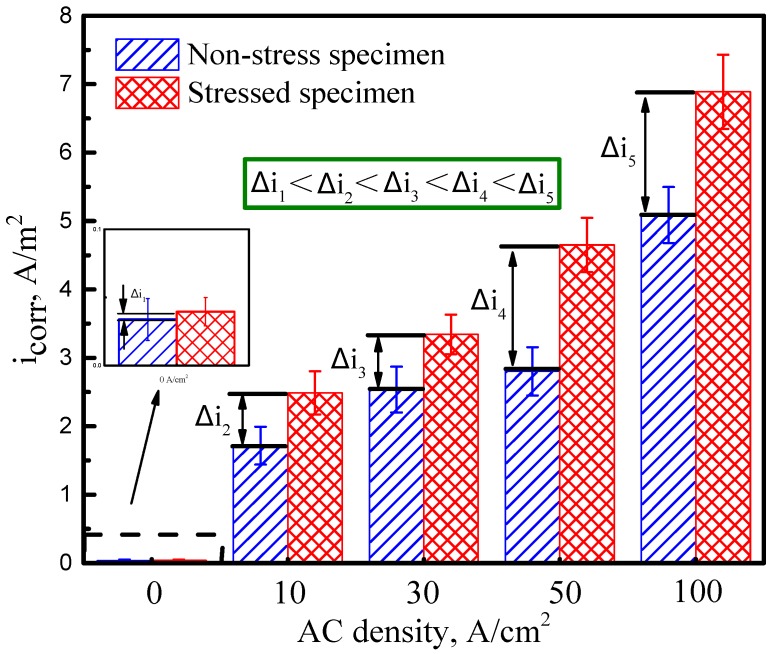
Corrosion current density of the X70 steel tested at various AC densities in simulated seawater.

Comparing Figure 4a with Figure 4b, it can be found that stress does not essentially change the mechanism of the electrochemical reaction of X70 steel with or without the interference of AC. As calculated by Tafel fitting, the *i*_corr_ of X70 steel tested at various ACs with or without stress is present in Figure 5. The fitted values indicate that whether the X70 steel is stressed or not, *i*_corr_ goes up with increasing AC densities. In addition, Figure 5 also illustrates that the difference of *i*_corr_ between the stressed and non-stressed specimen (i.e., Δ*i*) gradually enlarges with the increase in AC density. It can be concluded that there is a clear synergistic effect between AC and tensile stress, and thus SCC is likely to be accelerated.

### 3.2. Immersion Tests

**Figure 6:**
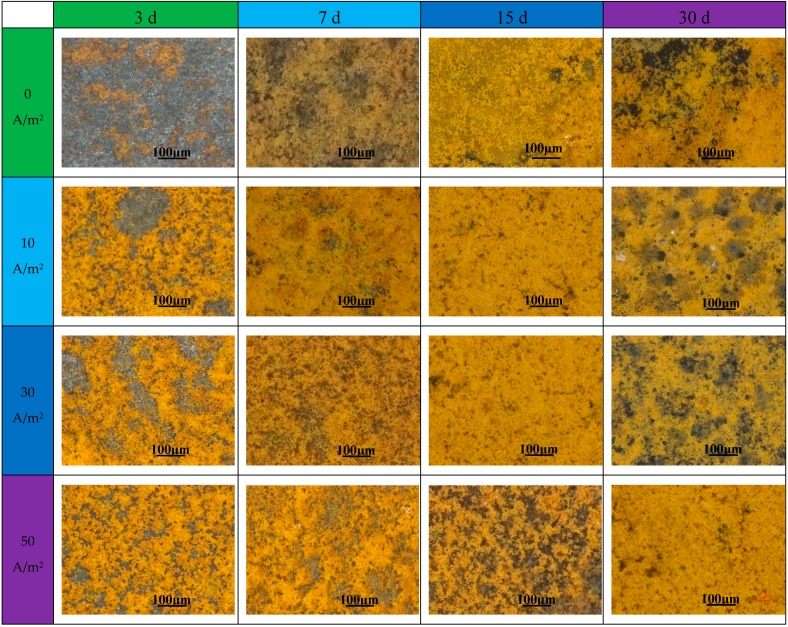

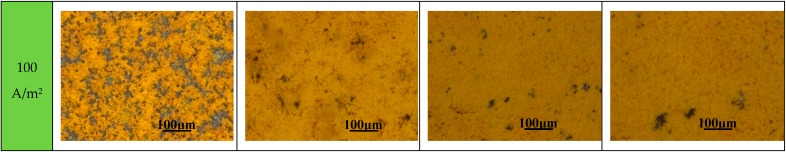
Optical view of the corrosion products on the tops of U-bend specimens after immersion for different time.

Figure 6 displays the optical morphologies of the corrosion products on the tops of U-bend specimens. It can be seen that after immersion for only three days, the specimens were badly corroded, especially with the interference of AC, and the higher the AC current density, the more the corrosion products were present. Clearly, the AD process was accelerated with the increase in AC. Besides, the surface color presented bright yellow for the corrosion products at the early stage. As time elapsed, corrosion products accumulated and gradually covered the whole surface; the surface also turned to dark yellow after immersion for 30 days. It is believed that localized corrosion may occur underneath the corrosion product film; in particular, stress corrosion may initiate at the top of U-bend specimens.

**Figure 7:**
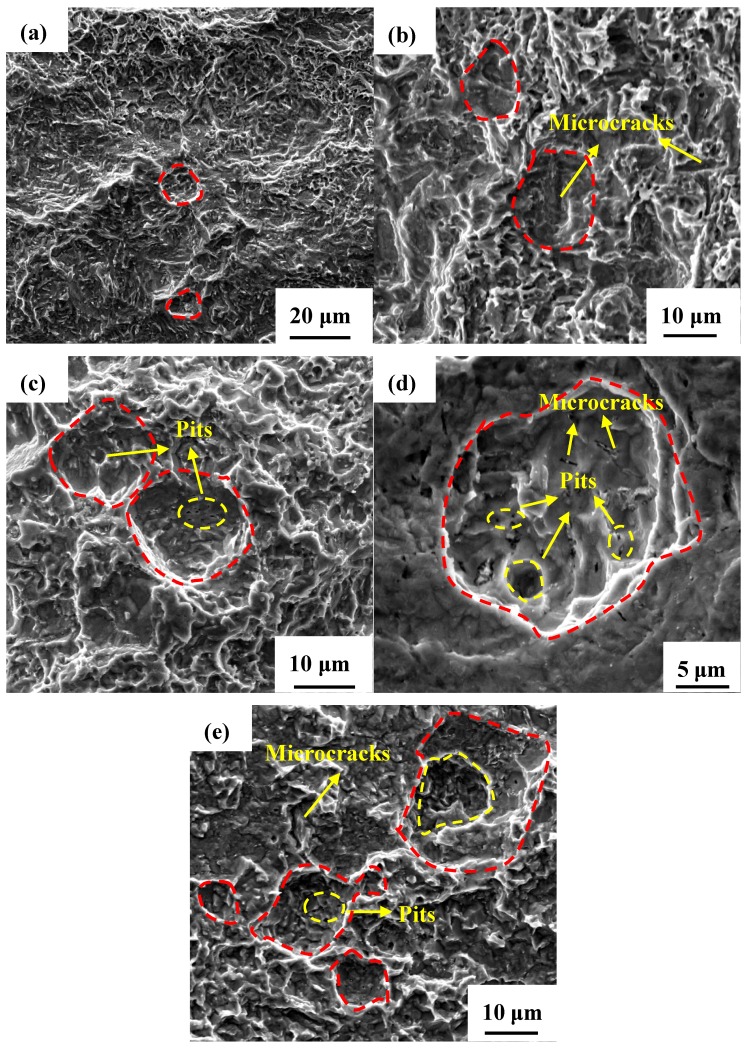
Surface morphologies of the U-bend samples immersed for 30 days in different AC densities: (**a**) 0 A/m^2^; (**b**) 10 A/m^2^; (**c**) 30 A/m^2^; (**d**) 50 A/m^2^; and (**e**) 100 A/m^2^.

Thus, the corrosion products were removed and the corrosion morphologies of the U-bend samples were carefully examined, as shown in Figure 7. The red dashed lines describe the visible localized corrosion areas on the top surface of U-bend specimens, and the yellow arrows and dashed lines represent the microcracks and pits initiating from these areas. It is indicated that severe corrosion occurs on the top of each U-bend specimen. Without AC, the specimen surface exerts general corrosion accompanied with few shallow pits, as shown in Figure 7a. However, when AC is applied, local AD occurs as pointed in Figure 7b–e, and its role in stress corrosion is significantly promoted on the top surfaces of specimens. Noting that with an increase in the AC density, pits extend and deepen. Besides, microcracks are also discovered in the bottom of pits. Obviously, the synergistic effect of stress and AC markedly accelerates the local AD process, which causes the initiation of microcracks and may eventually induce SCC.

### 3.3. SCC Behavior

#### 3.3.1. SSRT Curves and SCC Susceptibility

**Figure 8:**
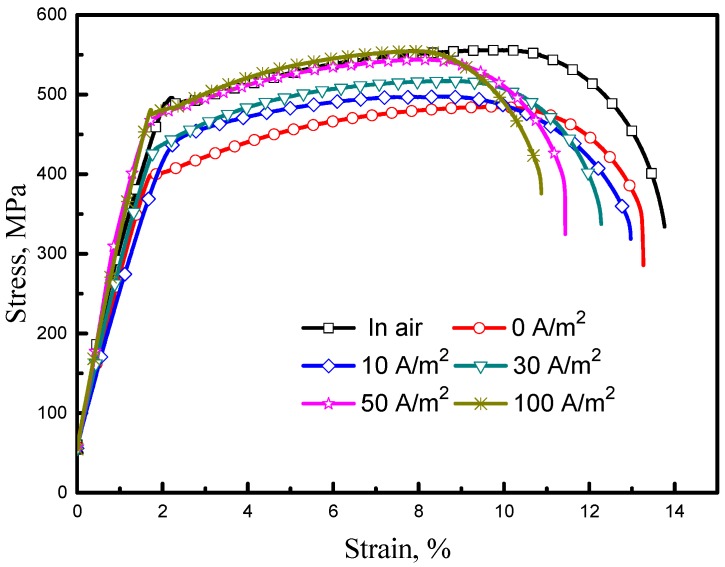
Stress–strain curves of the X70 steel after SSRT tests at various AC densities in simulated seawater.

**Figure 9:**
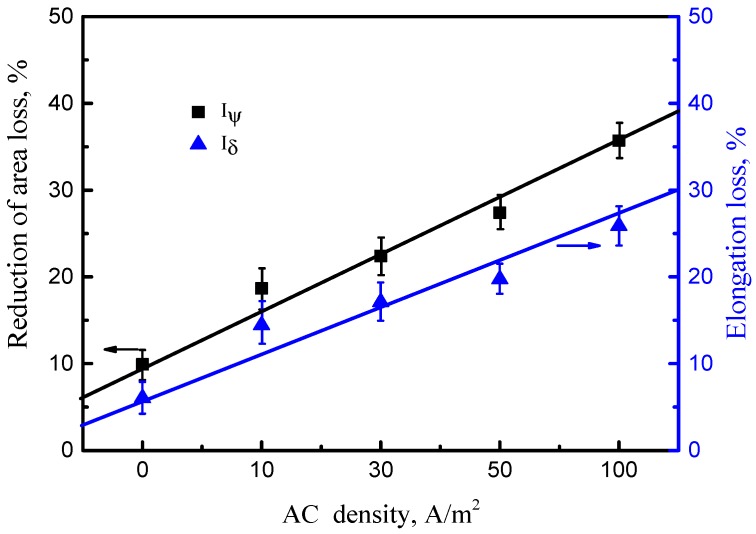
SCC susceptibility of the X70 steel at various AC densities in simulated seawater.

Figure 8 clearly shows the difference between stress-strain curves obtained by SSRT in air and solution with varying AC conditions. Most of the curves obtained in solution have lower tensile strength and shorter elongation than those in air, which implies SCC susceptibility. As the AC increases, the yield strength and ultimate-tensile strength increase, but the elongation rate decreases, which can be attributed to the influence of AC on cathodic current density. Under the interference of AC, hydrogen evolution turns to be the primary cathodic process, especially during the negative half wave stage, and the higher AC density results in higher hydrogen charging current density and more hydrogen content in steel. As published in previous papers [30,31], slight hydrogen in steel could impede the slip of dislocations, leading to an increase of yield strength of the steel. To compare the SCC susceptibility at different AC densities, *I*_Ψ_ and *I_δ_* were calculated and shown in Figure 9. It can be found that both *I*_Ψ_ and *I_δ_* rise with the increase in AC density, which corresponds to an increase in SCC sensitivity. More importantly, under the intervention of AC, the SCC susceptibility ratio increases by approximately four times, which indicates that AC can greatly enhance the SCC risk to offshore steel pipelines.

#### 3.3.2. Profile of Cracks and Their Propagation Modes

**Figure 10:**
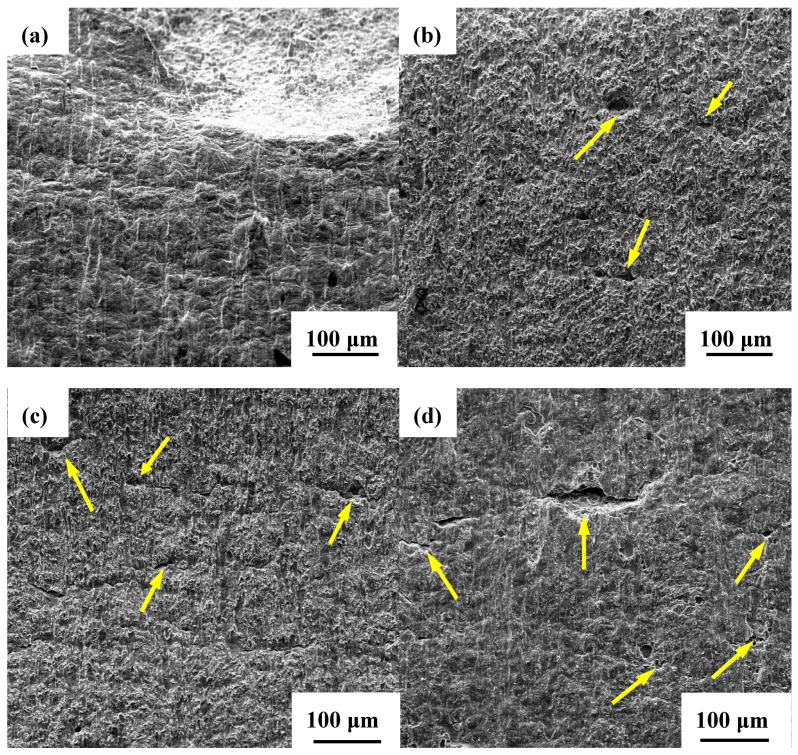

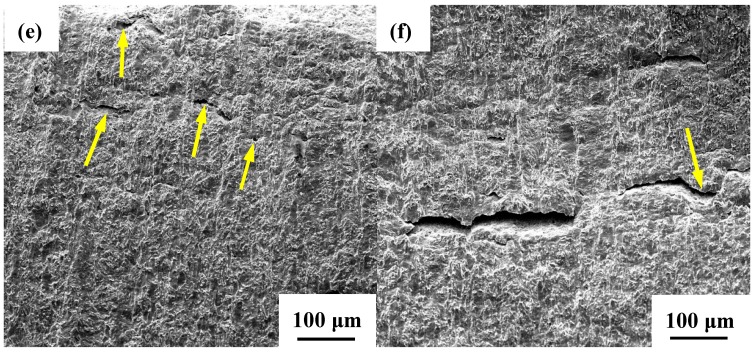
Side-surface morphologies of SSRT samples obtained at various conditions of (**a**) in air; (**b**) 0 A/m^2^; (**c**) 10 A/m^2^; (**d**) 30 A/m^2^; (**e**) 50 A/m^2^; and (**f**) 100 A/m^2^.

**Figure 11:**
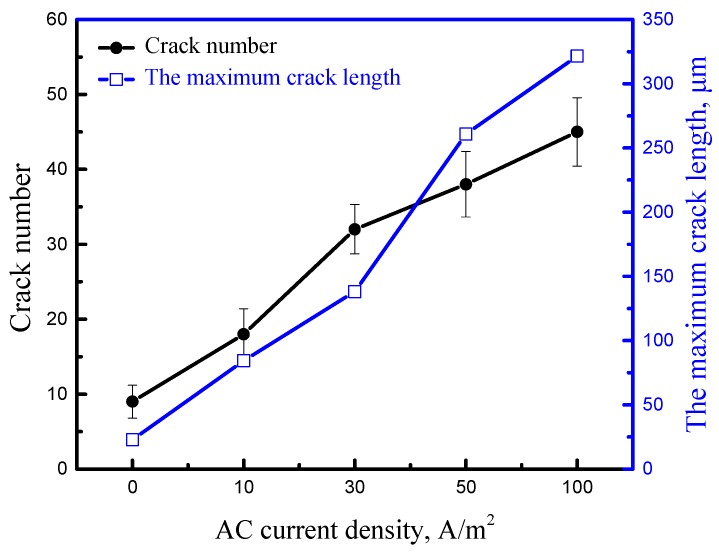
Number of cracks longer than 20 μm and the maximum crack length on the side-surfaces of SSRT specimens obtained at various AC densities.

The side-surfaces near the fracture of the SSRT specimens are observed and shown in Figure 10. It should be noticed that, compared to the morphology of the sample in air (Figure 10a), the quantity, length, and depth of the microcracks on the side-surfaces of the specimens increase as the AC density rises (Figure 10b–f). In order to better compare the crack information on the side-surfaces, the number of cracks longer than 20 μm is counted and the maximum crack length is also recorded in Figure 11. It can be clearly seen that both the crack number and the maximum crack length continually increase with the increasing AC density. Specifically, the number of cracks on specimens interfered with 100 A/m^2^ is quadruple of that without AC; moreover, the maximum crack length reaches up to 321.5 μm. Besides, the increase of the AC density aggravates AD process, because microcracks are apparently observed initiating from local AD sites under the different AC conditions, such as the locations marked with yellow arrows in Figure 10b–e. However, when the AC density rises to 100 A/m^2^, the size of the microcracks is much longer, while the degree of general corrosion around the cracks is reduced, as displayed in Figure 10f, which indicates that hydrogen may make a difference in SCC.

**Figure 12:**
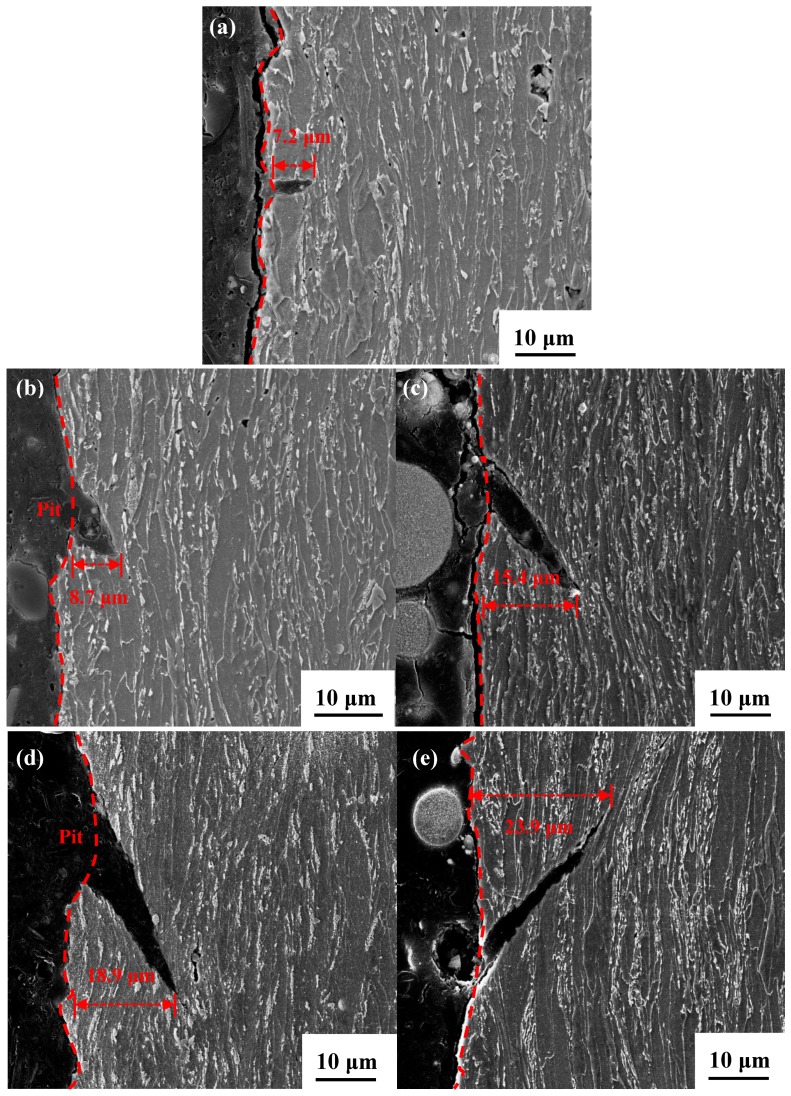
Cross-sectional fracture morphologies of the X70 steel at various AC densities of (**a**) 0 A/m^2^; (**b**) 10 A/m^2^; (**c**) 30 A/m^2^; (**d**) 50 A/m^2^; and (**e**) 100 A/m^2^ in simulated seawater.

For observing the SCC propagation mode, crack morphologies of the cross-section were observed, and crack depths are measured and displayed in Figure 12. Clearly, the cracks deepen with the enhanced AC density; the maximum value is 23.9 μm and appears at the specimen with the AC interference of 100 A/m^2^. Furthermore, under varying AC densities, cracks propagate transgranularly (TGSCC) in simulated seawater. The ends of the microcracks become sharper with the increase in AC density. This finding may explain the reason why the increase in the AC density facilitates the cathodic reaction and accelerates AD; the synergetic effect of AD and HE leads to the sharper and deeper cracks in TGSCC [32,33,34].

## 4. Discussion

### 4.1. Effect of AC on Electrochemical Reactions of X70 Steel in Simulated Seawater

Potentiodynamic curves can characterize the corrosion mechanism of X70 steel interfered by AC. In this paper, the electrochemical measurements consist of two parts: stressed and non-stress samples. Figure 4 reveals that stress can enhance the corrosion current density significantly but fail to vary the electrochemical mechanism. Therefore, it is reasonable to analyze the electrochemical mechanism according to polarization curves measured with stressed specimens (Figure 4b), the effect of AC on which is more obvious.

Obviously, both the anodic and cathodic processes are promoted by the application of AC; the shape of anodic curves does not change significantly, while the cathodic curves vary greatly. As shown in Figure 4, the cathodic curve presents a straight line with a slope angle of 45° in the absence of AC. In this case, oxygen is sufficient on the cathode, and the oxygen reduction reaction is shown in Equation (3). At the moment, the corrosion rate mainly depends on its velocity of discharge. However, once AC is applied, the cathodic curve exerts two parts including the vertical straight line at higher potentials and the crooked part at a lower potential. The former indicates concentration polarization due to the limited diffusion rate of oxygen, and the latter illustrates the occurrence of hydrogen evolution reaction after reaching the limiting diffusion current density of oxygen.

(3) $$O_{2}+2H_{2}O+4e\to 4OH^{-}$$

**Figure 13:**
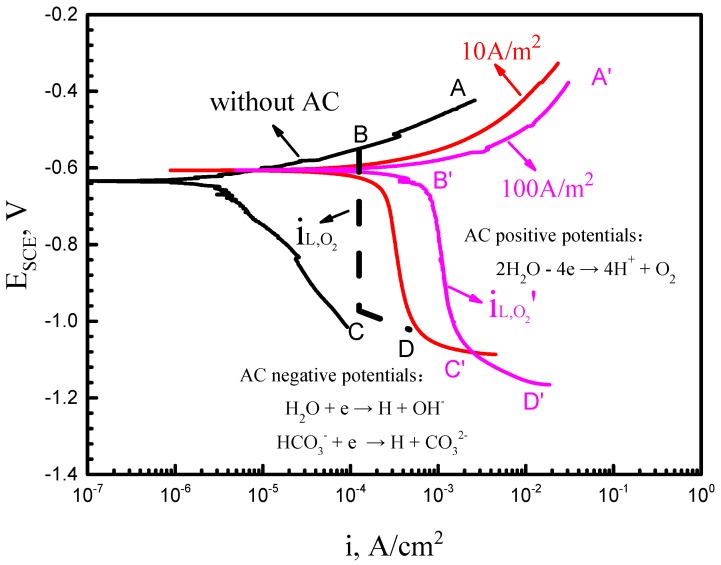
The effect of AC on the cathodic reaction of the X70 steel in simulated seawater.

This change of polarization curves indicates the transformation of the cathodic process, which is controlled by mass-transport process when AC is applied. Therefore, to decipher the effect of AC on the cathodic reaction, a theoretical curve is calculated and plotted in Figure 13, which is composed of the limit current of the diffusion of oxygen and the curve of hydrogen evolution. In a certain steady condition, the limiting diffusion current density is expressed as

(4) $$i_{L,O_{2}}=nFD\frac{C_{b}}{l}$$

In this equation, *n* is the charge number in oxygen reduction; *F* is the Faraday constant; *D* is the diffusion coefficient of oxygen in solution; *C_b_* is on behalf of the solubility of oxygen in solution and *l* is the thickness of the stagnant layer. *n*, *F*, *D*, and *C_b_* are all constant in the normal temperature and pressure experimental environment in this paper, i.e., *n* = 4, *F* = 9.65 × 104 C/mol, *D* = 1.98 × 10^−9^ m^2^/s, *C_b_* = 8.28 mg/L, where *D* and *C_b_* are approximated as diffusion coefficient and concentration of oxygen in pure water, respectively, for simplification; at room temperature, under the circumstance that only the natural convection of solution exists, the value of *l* after reaching stability is 10^−2^ cm [35]. Therefore, the limiting diffusion current density can be figured out: $i_{L,O_{2}}$ = 1.978 × 10^−4^ A/cm^2^.

The hydrogen evolution potential, *E_H_*’(SCE) is codetermined by the equilibrium potential, *E_H_^θ^*(SCE), and the overpotential, *η_H_*, which can be expressed by Equations (5) and (6):

(5) $$E_{H}{}^{\theta }(\mathrm{SCE})=-0.06\times \mathrm{pH}-0.241$$

(6) $$\eta_{H}=a_{H}+b_{H}\log i$$

where pH, *a_H_*, and *b_H_* are all constant; and in an alkaline environment, *a_H_* = 0.76 V, *b_H_* = 0.11 V; the corrosion current density can be fitted from Figure 4b, i.e., *I* = 3.96 × 10^−6^ A/cm^2^. The critical potential of hydrogen evolution reaction can be calculated as *E_H_*’ (SCE) = *E_H_^θ^* (SCE) − *η_H_* = −893 mV. Therefore, the established cathodic curve is shown as BCD in Figure 13. Apparently, with the application of AC, the part of BC presents a significant right shift, which shifts continuously with an increasing AC density. When the AC density reaches 100 A/m^2^ (B’C’), the order of magnitude of limiting diffusion current density is changed ($i_{L,O_{2}}\prime$ ≈ 1.15 × 10^−3^ A/cm^2^).

The above analysis demonstrates that AC affects cathodic reactions in the following two ways. When the AC cycle is in the positive half-cycle, the superposed potential during the electrochemical measurements is positive enough to motivate the electrolysis of water, resulting in the generation of O_2_ within the electric double-layer; the reaction is shown below:

(7) $$2H_{2}O-4e\;\to \;4H^{+}+O_{2}$$

The continuously produced oxygen accelerates the oxygen reduction process, as shown in Equation (3); cathodic current density increases intensively, and as a result, the cathodic current under AC is more than the limiting diffusion current density of oxygen. At the same time, the thickness of the stagnant layer, *l*, decreases with the increase in the AC density due to the acceleration of electromigration of ions, which will also shift $i_{L,O_{2}}$ positively. Thus, the corrosion current density and $i_{L,O_{2}}$ increase with AC density going up.

When the AC cycle is in the negative half-cycle, the transient potential on the surface of the electrode is much more negative than the hydrogen evolution potential, −893 mV, even lower than −2 V [36]. That is to say, hydrogen evolution can occur and accelerate the cathodic process. Under this circumstance, hydrogen atoms produced by the ionizations of water and HCO_3_^−^ (Equations (8) and (9)) rapidly participate in the cathodic reaction. The ionization reaction is swift and the reactant is abundant, so the hydrogen atoms can be generated at a steady flow, which is evidenced by the many bubbles forming on the electrode. As a result, the hydrogen evolution reaction plays a critical role in the corrosion of X70 steel and promotes the cathodic process in simulated seawater during the AC negative half-cycle.

(8) $$H_{2}O+e\to \;H+\;OH^{-}$$

(9) $$HCO_{3}{}^{-}+e\;\to \;H+\;CO_{3}{}^{2-}$$

### 4.2. Effect of AC on the Stress Corrosion Behavior of X70 Steel in the Simulated Seawater

In the absence of AC, active dissolution occurs on the surface of the X70 steel, which is electrochemically balanced by the underway depolarization. Hence, the corrosion process is in a stable state, leading to a relatively lower electrochemical reaction rate (Figure 4a). This finding indicates a less active level of the local AD and hydrogen evolution, which are acknowledged to be responsible for the lower sensitivity of formation of pits and cracks. Thus, SCC susceptibility is low (Figure 9). However, this situation is fully changed by the application of AC. Alternating current can result in the unsteady electrochemical state of X70 steel in simulated seawater through the cycle shift of positive and negative potentials. As reported in previous literature [37,38], the non-stable polarization process destroyed the local double electric layer, and the AD process during this case was accelerated not only under anodic potential but also in the cathodic potential range. As a result, the behavior and mechanism of the SCC shift significantly, which is primarily manifested in the following aspects.

On the one hand, AD effect is significantly improved throughout the whole cycle of AC. Firstly, for the positive half-cycle, active dissolution process and oxygenation reaction are both visibly promoted, as indicated by Figure 4, Figure 5 and Figure 12. Generally, the acceleration of oxygenation reaction can influence the growing rates of pits and cracks. Our study further indicates that the size of pits and cracks notably increases with the interference of increasing AC density; the arrows and dashed lines marked in Figure 7 and Figure 10 clearly point out this phenomenon. Specifically, the contribution of this influence can be significantly speeded up in orders of magnitude, as displayed in Figure 11. Thus, SCC susceptibility is enhanced surprisingly when AC is applied. Secondly, for the negative half-cycle, AD process still occurs due to the synergistic effect of the cyclic potential shift and local additional potential (LAP) effect [37,38]. Considering the unsteady electrochemical state in an AC cycle, even though the electrode potential becomes less negative and the reductive reaction is somewhat inhibited, the reductive reactions in defects may be more active than that in intact areas because of the LAP effect, especially under tensile stress [37]. As a consequence, an adequate driving force exists at the defect sites on the surface, and results in the localized AD effect in the negative-half-cycle of AC to promote pit initiation, as well as stress corrosion cracks. Furthermore, the local AD effect can be accelerated with the increase in AC density.

One the other hand, HE effect is also strengthened when the AC is in the negative-half-cycle. Hydrogen evolution occurs at this time, and cathodic charging current density continually improves with the increase in AC density. Thus, a continuous flow of H^+^ can be gathered on the surface of the specimen. Hydrogen ions can be changed into hydrogen atoms with a portion coming together and bubbling as H_2_. However, plenty of hydrogen atoms can permeate into the steel substrate by dislocations, vacancies, and other types of defects, thereby leading to the local distortion of stress, the degradation of mechanical properties of the steel, and the facilitation of the initiation and propagation of microcracks [39,40,41,42]. In addition, the permeation of hydrogen atom leads to increased numbers of defects in the steel substrate. In this way, hydrogen induced anodic dissolution (HIAD) is likely to happen under the unstable cathodic polarization in near-neutral pH environments, therefore resulting in the formation of cracks and eventually causing SCC. A rise in the AC density causes the aggravation of the hydrogen evolution reaction, so there is an increased risk of HE or HIAD. The crack initiation and propagation processes are accelerated, and the SCC susceptibility is also improved. Above all, the risk of SCC failure in seawater is greatly increased due to the synergistic effect of AD and HE introduced by AC.

## 5. Conclusions

In the presence of AC, the electrochemical mechanism of X70 steel in simulated seawater is altered. With the increase in AC density, the oxygen reduction process is accelerated, and its limiting diffusion current density, namely, $i_{L,O_{2}}$, is significantly improved. Particularly, hydrogen evolution reaction is motivated even in a neutral seawater environment under the interference of AC, which further facilitates the cathodic process. Accordingly, corrosion current density is immensely enhanced, and corrosion resistance is worsened.

In the presence of AC, to a certain extent, SCC susceptibility appears on the X70 steel in simulated seawater. In addition, SCC susceptibility sharply increases with the increase in AC current density. With the alternating action of the positive and the negative half cycle of AC, hydrogen generated from the solution permeates into the substrate and notably affects SCC, and the effect of AD is also markedly strengthened due to the accelerated oxygenation reaction and LAP effect. Therefore, the SCC of X70 steel is controlled by a mixed mechanism of AD and HE, with a TGSCC mode.
